# The Influence of Dual Source Cerebellar Transcranial Direct Current Stimulation on Muscle Fatigue

**DOI:** 10.3390/jfmk11030320

**Published:** 2026-08-18

**Authors:** Janriek A. Bognot, Eliza Clinton, Erik W. Wilkins, Richard J. Young, Mina Rezaeian Jam, Ryder Davidson, George Alhwayek, Reese Krider, Jonathan A. Park, Zachary A. Riley, Brach Poston

**Affiliations:** 1 Department of Kinesiology and Nutrition Sciences, University of Nevada-Las Vegas, Las Vegas, NV 89154, USA; bognot@unlv.nevada.edu (J.A.B.); parkj62@unlv.nevada.edu (J.A.P.); 2 Interdisciplinary Ph.D. Program in Neuroscience, University of Nevada-Las Vegas, Las Vegas, NV 89154, USA; clinte1@unlv.nevada.edu (E.C.); erik.wilkins@unlv.edu (E.W.W.); ryoung@unlv.edu (R.J.Y.); 3 Department of Computer Science, University of Nevada-Las Vegas, Las Vegas, NV 89154, USA; rezaem2@unlv.nevada.edu; 4 School of Medicine, University of Nevada-Las Vegas, Las Vegas, NV 89154, USA; davidr6@unlv.nevada.edu (R.D.); alhwag1@unlv.nevada.edu (G.A.);; 5 Department of Kinesiology, Indiana University Indianapolis, Indianapolis, IN 46202, USA; zariley@iupui.edu

**Keywords:** transcranial direct current stimulation, muscle fatigue, cerebellum, non-invasive brain stimulation, force variability, electromyography

## Abstract

**Background:** Transcranial direct current stimulation (tDCS) applied unilaterally to the cerebellum (c-tDCS) can improve several aspects of human motor performance. The purpose of the study was to examine the influence of dual source tDCS delivered bilaterally over the cerebellar cortices (dsc-tDCS) on the time to task failure (TTF) of a fatiguing contraction in young adults. **Methods**: The study utilized a double-blind, randomized, SHAM-controlled, within-subjects, crossover design, and participants were given either dsc-tDCS or SHAM stimulation in two different experiments held 7 days apart in a fully counterbalanced order. Every aspect of the two experiments was the same except the type of stimulation (dsc-tDCS or SHAM) delivered during the fatiguing contraction. The fatiguing contraction was executed with a precision grip at 15% of the maximum voluntary contraction (MVC) force, and participants were instructed to maintain the contraction for as long of time as possible. **Results**: The TTF and fatigue index were both similar for the dsc-tDCS and SHAM stimulation conditions. In addition, the electromyographic (EMG) activity, force error, and standard deviation (SD) of force measured during the fatiguing contraction were also not statistically different between the dsc-tDCS and SHAM stimulation conditions. **Conclusions:** The findings suggest that dsc-tDCS does not decrease the rate of progression of muscle fatigue.

## 1. Introduction

Muscle fatigue is typically defined as an impermanent reduction in the maximum force-generating ability of muscle due to exercise [1,2,3,4,5,6,7,8,9]. In almost every physical task, muscle fatigue emerges due to some combination of physiological factors that originate at the central nervous system level or the muscular level [1,5,10,11], which are respectively referred to as central fatigue and peripheral fatigue. Accordingly, the relative contribution of central and peripheral processes to fatigue development critically depends on the details of the motor task being performed [3,4,7,11,12]. In sustained submaximal isometric contractions, for example, it has been estimated that physiological changes that occur at the cortical level are responsible for approximately one half to two-thirds of the total fatiguability exhibited in these circumstances [5]. This would imply that modalities that act on cortical areas could theoretically have the greatest potential to reduce the rate of progression of muscle fatigue during submaximal muscle contractions.

Although many of the physiological mechanisms that promote the development of muscle fatigue have been well-known and studied for a long time, there is a limited number of mitigation strategies available to slow the progression of muscle fatigue [13]. This is important because muscle fatigue has detrimental influences on all motor abilities and every facet of human performance (e.g., maximal force, movement accuracy, movement variability, etc.), especially in various movement disorders and patient populations. Similarly, the primary methods available to address the progression of muscle fatigue, such as training methodologies, diet strategies, and nutritional supplements, are already well-known and are in widespread use. Furthermore, many of these interventions are only efficacious in certain situations, environments, or types of motor tasks [13]. Accordingly, new intervention methods capable of complementing existing fatigue mitigation strategies would have numerous important practical implications [8,13,14].

Basic logic and theoretical considerations would suggest that to reduce the rate of progression of muscle fatigue, a modality would have to exert effects on one or more of the primary physiological mechanisms that contribute to the development of muscle fatigue during a given motor task. The physiological adjustments that constrain the ability to maintain a fatiguing contraction have been described in the most detail in submaximal isometric contractions, although in general the same adjustments most likely also occur to varying extents in anisometric contractions. One major change in motor output during submaximal fatiguing isometric contractions is a decline in discharge rate of some portions of the motor units activated at the start of the task [1,6,15], which in isolation would lead to decreases in the force that could be produced. However, this adaptation is compensated for through the recruitment of additional motor units that allows the requisite target force to be maintained and the contraction to continue for some period of time [1,5,6,7]. The gradual recruitment of initially inactivated larger higher-threshold motor units results in enhancements in force error and standard deviation (SD) of force (reductions in force accuracy). In addition, another prominent physiological change that occurs during fatigue, which significantly influences the TTF, is the enhanced feedback delivered by group III and IV afferents. These afferents are dispersed throughout muscles and respond to various by-products of muscle metabolism, including potassium ions, lactate, arachidonic acid, bradykinin, and several others, as well as phenomena such as pain and temperature. In general, increased group III and IV afferent activation during a fatiguing muscle contraction can inhibit net motor output via presynaptic inhibition of Ia afferents, direct inhibition of motor neurons [1], and inhibition manifesting at the level of the cortex [4,5,16,17].

Transcranial direct current stimulation (tDCS) is one of the few possible interventions that could target several of the physiological factors that collectively contribute to the progression of muscle fatigue. For instance, numerous studies have shown that tDCS can significantly increase motor skill and motor learning in simple and complex motor tasks [18,19,20,21,22,23,24]. This has been most commonly demonstrated when anodal tDCS is applied unilaterally to M1 or the cerebellum (M1-tDCS and c-tDCS) for a duration of 10 to 20 min before or during practice of a motor task [18]. When compared to SHAM stimulation (task practice alone), tDCS of these brain areas usually results in acute motor skill enhancements of 10–15%. In addition, M1-tDCS also usually leads to increases in M1 excitability on the order of 20–40% in these circumstances. Although not as numerous as motor skill studies, a significant number of investigations have also found that tDCS can prolong endurance time or the time to task failure (TTF) of various motor tasks [9,25,26,27,28,29]. These results are thought to occur due to some combination of the ability of tDCS to improve motor skill, M1 excitability (descending drive), and pain tolerance [30].

Most of the aforementioned muscle fatigue studies have delivered tDCS unilaterally to the dorsolateral prefrontal cortex (DLPFC) or M1 during either a sustained isometric contraction of proximal upper limb muscles [28,29,31] or in lower body cycling tasks [32]. However, accumulating evidence has also shown that c-tDCS can not only improve motor skill in both single and multi-joint tasks [18,19,20,23,24], but also may enhance motor qualities such as maximal force production [33]. For motor skill studies, the most effective c-tDCS electrode montage has involved unilateral stimulation with the anode placed over the right cerebellum and the cathode over the ipsilateral buccinator muscle to improve performance of the right hand and arm system. Nonetheless, other studies have found significant tDCS effects utilizing other electrode montages. For example, Kenville et al. [33] reported that c-tDCS delivered bilaterally with a moderately sized single anode and a large cathode on the right buccinator muscle increased the maximal voluntary contraction (MVC) force of an isometric barbell squat exercise. More recently, bilateral tDCS of different brain areas has been accomplished utilizing novel dual source stimulation protocols involving either a dual-channel or two separate stimulation units to concurrently target bilateral homologous regions of the brain. Accumulating evidence suggests that these dual or bilateral montages may be more effective compared to single-site electrode montages [34,35,36,37]. In a comprehensive study, Anoushiravani and colleagues (2023) compared the abilities of dual source tDCS delivered bilaterally to the premotor cortices, dual source bilateral c-tDCS (hereafter termed dsc-tDCS), and SHAM stimulation to improve measures of motor coordination, force production, muscle endurance, and muscle activation during complex motor tasks in gymnasts [38]. The findings indicated that bilateral premotor tDCS enhanced muscle power and strength, whereas dsc-tDCS improved a motor task that involved a combination of muscle strength, muscle endurance, and multi-joint coordination compared to SHAM stimulation. Furthermore, dsc-tDCS increased the amount of muscle activation as measured by surface EMG activity of a number of upper body muscles. These results are particularly impressive because they were attained in trained athletes performing complex motor tasks that involved not only motor skill, but also high levels of muscle force production. Furthermore, if dsc-tDCS can improve these motor abilities in healthy young adults and trained athletes where ceiling effects can occur, it would have a high probability of success in improving motor function in rehabilitation settings and in populations such as older adults [39], various movement disorders [40], and in cerebellar patients [41].

The purpose of the study was to examine the influence of dsc-tDCS on the TTF of a fatiguing contraction in young adults. Based on the findings of the aforementioned recent study [38] that reported improved strength, muscle endurance, and muscle coordination in gymnasts following dsc-tDCS, it was hypothesized that dsc-tDCS would prolong the TTF of a fatiguing contraction performed by hand muscles compared to SHAM stimulation. Furthermore, it was expected that the percentage decline in pre- to post-MVC force (fatigue index) would be lower for the dsc-tDCS condition. Thus, it was hypothesized that the rates of rise in EMG, force error, and SD of force would be greater in the SHAM condition compared to the dsc-tDCS, which would all contribute to the shorter TTF in the SHAM condition. These hypotheses were not only based on the aforementioned c-tDCS studies and the dsc-tDCS study that found increases in motor skill and force production, but also on studies showing that have shown that the cerebellum is involved in perception of physical effort and fatigue [42], pain [43,44], sensorimotor integration [40,45,46], error correction [40,45,46,47], and visuomotor control [48]. In the current study, a sustained submaximal isometric contraction task paradigm was used, as this is the most commonly used experimental arrangement in fatigue studies because it allows for strict experimental controls and facilitates measurement of physiological variables. In addition, a precision grip force matching task was used, as this task is commonly used in fatigue and motor control studies and involves several aspects of cerebellar function, such as visuomotor control [48], error correction (around the target force level), and precision grip control [48].

## 2. Materials and Methods

### 2.1. Participants

Twenty healthy adult participants (12 males, 8 females; average ± standard deviation age: 28.00 ± 7.96 years) were recruited for the study. Participants were all active young adults, but none were currently competitive athletes (e.g., participating in a college sport). The nature, benefits, and risks of all experimental procedures were explained to the volunteers, and their written informed consent was acquired before participating in the study. All participants were right-handed as indicated by the laterality index (0.93 ± 0.10) of the Edinburgh Handedness Inventory [49] and were free from neurological disorders, psychiatric conditions, and uncontrolled medical conditions. Participants were excluded if they were pregnant or thought to be pregnant or previously had concussions, migraines, or seizures. Furthermore, participants were screened to verify that they did not meet the tDCS or transcranial magnetic stimulation (TMS) exclusion criteria [50,51]. All procedures were reviewed and approved by the University of Nevada Las Vegas Biomedical Institutional Review Board and conducted according to the Declaration of Helsinki.

### 2.2. Experimental Protocol

The study utilized a double-blind, randomized, SHAM-controlled, within-subjects, crossover experimental design that required participants to complete two separate experiments conducted a week apart [52,53,54] in a fully counterbalanced order. Every aspect of the two experiments was the same except the type of stimulation (dsc-tDCS or SHAM) delivered during the fatiguing contraction. The presentation order of the dsc-tDCS or SHAM conditions given to participants was randomized (Research Randomizer; www.randomizer.org; accessed on 15 January 2024) by a member of the research team that was not involved in the data collection during the experiments.

### 2.3. Experimental Procedures

**Figure 1:**
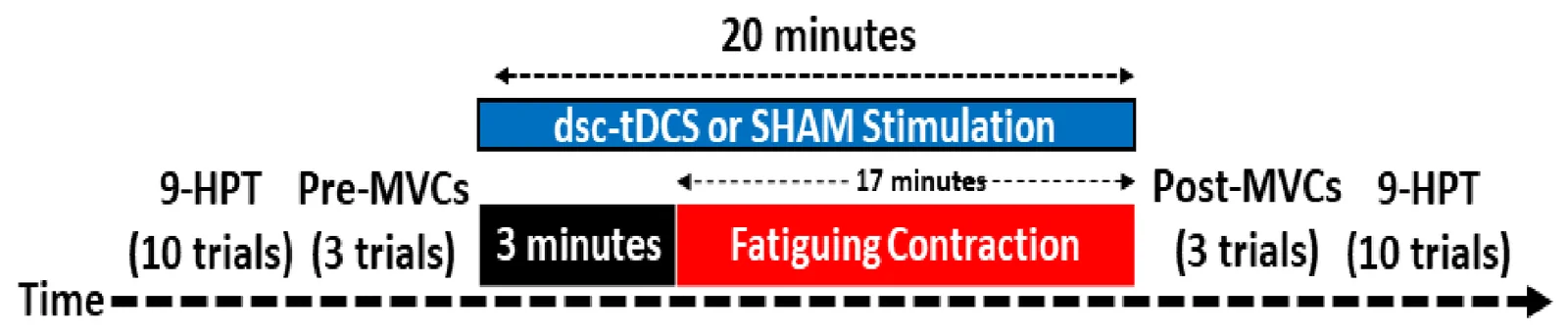
Schematic illustration of the experimental protocol. All participants completed two experimental sessions involving a fatiguing contraction in counterbalanced order, with one session involving dsc-tDCS application and one session involving SHAM stimulation. The 9-HPT and MVCs were performed before and after the fatiguing contraction and stimulation portion of the experiment in both sessions.

Each experiment involved the following six experimental procedures: (1) the nine-hole peg test (9-HPT); (2) pre-MVCs; (3) electrode montage placement; (4) application of either dsc-tDCS or SHAM stimulation for three minutes immediately followed by 17 more minutes of stimulation simultaneous with the execution of the fatiguing contraction; (5) post-MVCs; and (6) 9-HPT (Figure 1).

#### 2.3.1. 9-HPT Assessment

The Rolyan 9-HPT was used in the current study as a motor skill transfer task performed under fatigue. Accordingly, it was executed at the beginning of each experiment in a non-fatigued state and at the end of each experiment immediately after the fatiguing contraction and MVCs in a fatigued state. Thus, if dsc-tDCS were to improve motor skill during the fatiguing contraction (force error), a generalization of motor skill improvement under fatigue to the 9-HPT would provide further evidence that dsc-tDCS exerted some of its effects through motor skill enhancements. The 9-HPT is a common measure of manual dexterity [55], a component of the National Institutes of Health (NIH) motor battery toolbox [56], and also uses a precision grip. The 9-HPT was conducted according to standard procedures and involved grasping and moving 9 pegs one at a time from a dish to holes in a pegboard and back to the dish as quickly as possible [57]. This sequence of steps was repeated for a total of 10 trials for the 9-HPT assessments performed both at the beginning and end of the experiments.

#### 2.3.2. EMG Measurement

Surface EMG signals were measured from the first dorsal interosseous (FDI) muscle of the right hand during the MVCs and the fatiguing contraction using two electrodes configured in a belly-tendon montage. This was accomplished using Red Dot, Neonatal, Pre-Wired disposable electrodes (3M Company, St Paul, MN, USA) and Cambridge Electronic Design (CED) hardware (1902 Amplifier and Micro 1401 analog-to-digital converter; Cambridge, UK) for all experiments.

#### 2.3.3. MVC Measurement

All force data in the experiments were collected using two identical force transducers (Model S215; Strain Measurement Devices; Meriden, CT, USA), which were permanently mounted on both sides of a custom-designed grip manipulandum placed on a table surface. This allowed for the index finger and thumb to perform the same precision grip for both the MVC and fatiguing contraction tasks. For the MVCs, participants sat comfortably in a chair beside the grip manipulandum and table. The right arm was abducted to an angle of ~45° with the forearm resting on the table surface. The elbow joint angle was set to ~90°, the wrist was in a neutral position, and the hand was semi-supinated. The index finger and thumb performed the precision grip MVC task using similar methodology as prior studies [52,58]. The participants were instructed to produce the maximal force possible as quickly as possible and to hold the maximum for five seconds for each of the MVCs [59]. Three MVCs were completed before the fatiguing contraction, and three MVCs were completed immediately after the fatiguing contraction (termed pre- and post-MVCs, respectively). The investigators enforced a one-minute rest period between all MVC trials. Importantly, the MVC with the highest force among the three pre-MVCs was taken and utilized to calculate target force (15% of MVC) for the fatiguing contraction performed in each experimental session. Finally, the first of the three post-MVCs was conducted within a few seconds immediately after the fatiguing contraction ended.

#### 2.3.4. dsc-tDCS

Two separate NeuroConn DC Stimulators (Jali Medical; Framington, MA, USA) were used to deliver anodal tDCS bilaterally to the left and right cerebellar cortices with four rubber electrodes (5 × 7 cm) placed in saline-soaked sponges. The electrode montage and stimulation parameters were the exact same as those utilized by Anoshiravani et al. [38]. Briefly, the anode and cathode electrode pair from each stimulator was placed on the cerebellum and supraorbital region on one side of the body. The anode electrode placements were determined utilizing the 10–20 International EEG System O9 and O10 positions, whereas the cathodes were placed over the Fp1 and Fp2 positions. The electrodes were all orientated with the 7 cm sides in the anterior to posterior direction [38]. The stimulation intensity (current strength) was 2 mA for each stimulator, and the stimulation was delivered for up to 20 min depending on the TTF achieved by a given participant in each experiment. Finally, SHAM stimulation was delivered using standard procedures in the field [52,58] and consisted of a current ramp of 10 s, maintenance of the current at 2 mA for 30 s, and a current ramp down over 10 s for each of the two stimulators.

The application of the dsc-tDCS relative to the fatiguing contraction is depicted in Figure 1 and proceeded in three steps: (1) both tDCS devices were programmed with a stimulation duration of 20 min. Specifically, the first three minutes of stimulation were delivered while the participant was at rest [52]; (2) participants were required to start the fatiguing contraction at the 3 min mark while the stimulators continued to deliver current; and (3) the current was kept on for up to 17 min (20 min total) or until task failure, at which point the stimulators were quickly turned off by one of the investigators. Thus, the actual stimulation time varied to some extent across each participant and across each of the two experimental sessions [28,29], but can be determined for each condition by taking the TTF times reported in Section 3 and adding 3 min to the respective values. The delivery of current through the two stimulators was accomplished by a member of the research team who did not partake in data collection as in prior studies [52,58,60]. Finally, the investigators who completed the data collection in the experiments were blinded to the experimental condition given in each of the two experimental sessions.

#### 2.3.5. Fatiguing Contraction Task

The general experimental paradigm for the sustained isometric fatiguing contraction using the precision grip task was identical to a previous fatigue study [52]. The fatiguing contraction entailed grasping the manipulandum with a precision grip so that the center of the figure pads of the index finger and thumb were over the center of their respective force transducers on either side of the manipulandum. The target force was set to 15% of the pre-MVC force value for each individual participant and in each of the two experimental sessions. The instructions were to match the force produced to the 15% target force line as accurately as possible and for as long as possible until task failure. Thus, the total time the requisite target force could be maintained in the fatiguing contraction was denoted as the TTF. This was accomplished using continual online visual feedback of a red force trace that represented the precision grip force relative to the black horizontal target force line. Accordingly, the red force trace that was produced was superimposed on the computer screen with the target force line. Furthermore, another black horizontal line was placed on the monitor at a force level of 90% of the target force line. This line served as part of the task termination criteria, and participants were instructed to keep their force above this second line at all times and that any fall below the line should be corrected immediately by increasing the force produced to go back to the target line. There were three termination criteria for the fatiguing contraction [52,61] which included: (1) a decline in force below the 90% of target force threshold for more than 3 consecutive seconds; (2) a lack of ability to maintain the correct body or hand and arm posture despite strong verbal encouragement; and (3) a voluntary end to the fatiguing contraction due to an inability to continue holding the target force line, despite the best efforts of the participant. Similar to most studies involving sustained isometric fatiguing contractions, this was the most common way that the fatiguing contraction was terminated [52].

### 2.4. Data Analysis

The force and EMG data in the experiments were recorded using the Signal software Version 5.04 system (Cambridge Electronic Design, Cambridge, UK). For offline data analysis, custom scripts in Signal and in the Python 3.8 (Fredericksburg, VA, USA) programming language were utilized. The primary dependent variables were the TTF of the fatiguing contraction and the fatigue index. The TTF was calculated as the time in seconds that the fatiguing contraction was maintained until one of the criteria for task failure occurred. The fatigue index was quantified as the percent change in force between the pre-MVC and the first post-MVC conducted following the fatiguing contraction [1]. Specifically, the fatigue index was calculated as the post-MVC value minus the pre-MVC value, divided by the pre-MVC value, and multiplied by 100. Finally, although all participants showed a decline in MVC force from the pre-MVC to post-MVC, the absolute value of this result was taken to make all the percent change numbers positive for easier presentation.

Secondary dependent variables included the pre-MVC force and the target force. As mentioned previously, the pre-MVC was denoted as the MVC trial with the highest force amongst the three pre-MVCs, whereas the target force was calculated and set to 15% of the pre-MVC force. Additional secondary dependent variables that were collected during the fatiguing contraction included the average force (*a*force), average EMG (*a*EMG), force error, and SD of force. These variables were calculated over four equal time epochs, E1, E2, E3, and E4, which comprised time segments of 25% of the duration of the fatiguing contraction in each experiment. Specifically, the *a*force was calculated as the mean force generated in each of the four epochs. The *a*EMG was analyzed by taking the right FDI interference EMG signal and processing it by removing the DC offset, subjecting it to full-wave rectification, and normalizing it to the pre-MVC maximum rectified EMG [62]. Lastly, the average rectified EMG recorded in each time epoch was denoted as the *a*EMG and used for analysis. The force error was calculated in the same manner as previous fatigue and motor skill studies [52,58,59]. Briefly, the absolute value of the difference in the force generated relative to the target force was calculated for each sampling point and subsequently averaged over each of the four epochs. For SD of force, it was simply calculated as the SD of the force signal for each of the four time epochs that comprised the fatiguing contraction. Finally, the secondary outcome measure of 9-HPT performance was quantified as the time to complete the placement and replacement of the pegs, and the 10 trial averages were used for analysis.

### 2.5. Statistical Analysis

The dependent variables of TTF, fatigue index, pre-MVC, and target force were compared between the dsc-tDCS and SHAM conditions using separate two-tailed paired *t*-tests. Despite the fully counterbalanced design and the use of the most common washout period in tDCS studies, the possibility of an order effect or differential order effect for the presentation of the conditions was evaluated statistically for the primary outcome measure of TTF. This was accomplished by making order a between-subjects factor in an additional statistical analysis. Accordingly, a mixed 2-condition (dsc-tDCS, SHAM) × 2-order (1, 2) ANOVA with the factor condition being within-subjects and the factor order being between-subjects was used to rule out this possibility for TTF. The dependent variables of *a*force, *a*EMG, force error, and SD of force were compared between the dsc-tDCS and SHAM conditions and across the four epochs with separate 2-condition (dsc-tDCS, SHAM) × 4-epoch (E1, E2, E3, E4) within-subjects ANOVAs. In contrast, 9-HPT times were compared between the dsc-tDCS and SHAM conditions and between the two tests using a 2-condition (dsc-tDCS, SHAM) × 2-test (pre, post) within-subjects ANOVA. When Mauchly’s test of sphericity was violated, the Greenhouse–Geisser correction was used when appropriate. The level of significance was set to *p* < 0.05 for all statistical tests, except when adjusted by Bonferroni post hoc corrections if appropriate. The effect sizes are given as Cohen’s *d* (*t*-tests) and partial eta squared (η_p_^2^) for the ANOVAs. Data are reported as the means ± the standard errors in the figures and as the means ± the standard deviations in Table 1.

After the completion of data collection and analysis of the 20 participants, it was decided to conduct a futility analysis in a manner analogous to our prior studies [52,54,58,63] and to methods used in clinical trial research. This was undertaken to establish if further enrollment of participants and additional expenditure of research resources were needed or reasonably justified. Furthermore, we wanted to determine in a rigorous statistical manner whether the null findings represented a true lack of dsc-tDCS effects. Therefore, the means and SDs for the TTF and fatigue index for the dsc-tDCS and SHAM conditions reported in Section 3 below were utilized to establish the sample size that would be required to attain sufficient power to find statistically significant differences in the specific experimental methodological conditions of the current study. G*Power version 3.1.9.7 software and the associated module “Test family: *t* tests, the Statistical test Means: Difference between two dependent means (matched pairs) were used with the input parameters of a two-tailed test, α err prob = 0.05, and power (1-β err prob) = 0.80. The results revealed that 144,968 participants (144,948 additional participants) would be required to attain sufficient power to find significance for TTF. Similarly, the same processes revealed that 123 participants (113 additional participants) would be needed to achieve sufficient power to find significance for the fatigue index. Thus, these futility analyses for the two primary outcome measures were in overall general agreement and clearly indicated that it was very unlikely that the lack of significant differences between the dsc-tDCS and SHAM conditions was a result of the sample size in the current study. Based on the obvious impracticality of the enrollment of this number of additional participants, along with the very high *p* values and low effect sizes reported in Section 3 below, we chose to stop this study for futility after 20 participants. Overall, the futility analyses indicated that there were no meaningful treatment effects elicited by dsc-tDCS in the specific experimental conditions of the current study.

## 3. Results

### 3.1. Pre-MVC and Target Force

**Figure 2:**
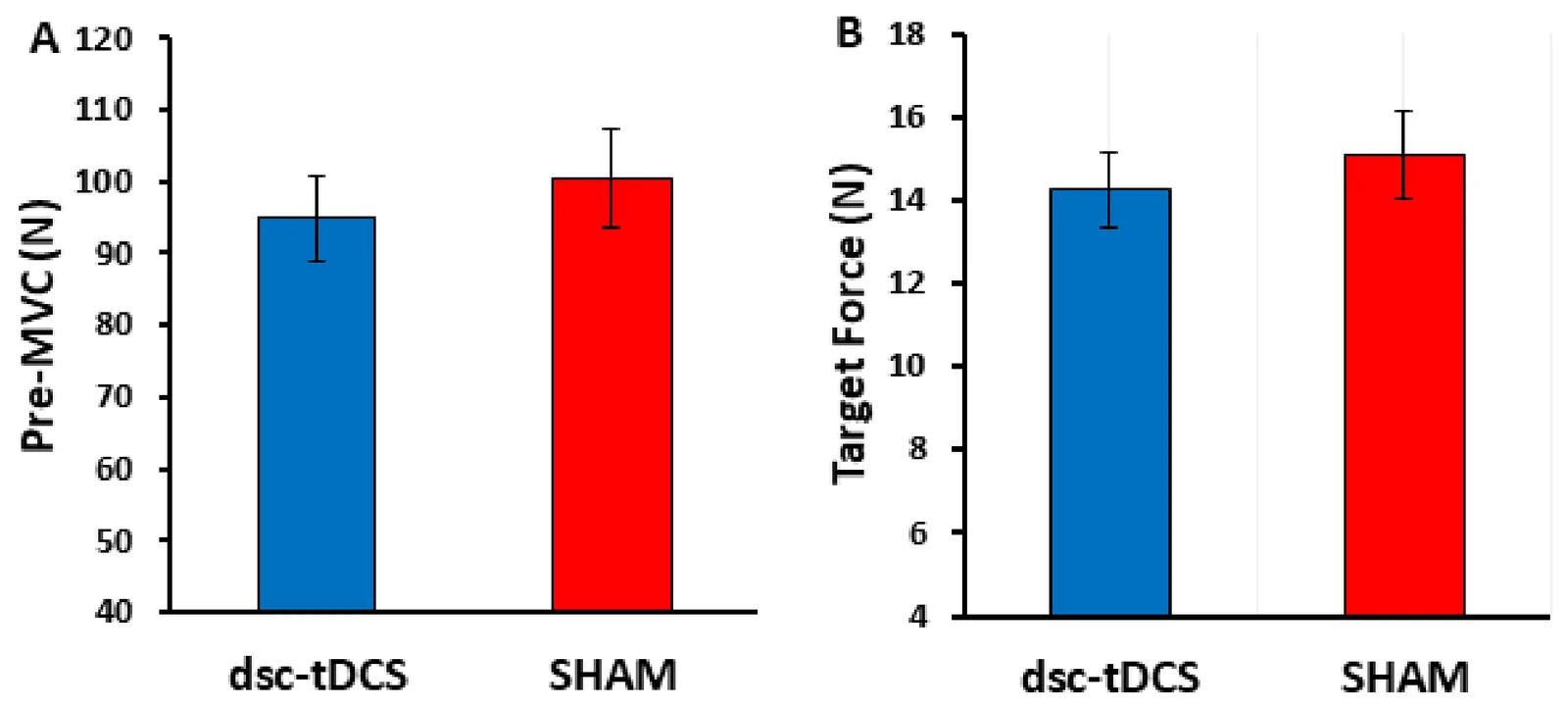
(**A**) The pre-MVC values for the dsc-tDCS and SHAM stimulation conditions. The pre-MVC values were similar for the two conditions. (**B**) The resulting target force values for the dsc-tDCS and SHAM stimulation conditions. The target force values were similar for the two conditions as they were set at 15% of the pre-MVC value for each condition.

The paired *t*-tests indicated that the pre-MVC (*p* = 0.149, *d* = 0.336, Figure 2A) and therefore the target force (*p* = 0.149, *d* = 0.336, Figure 2B) were not statistically different between the dsc-tDCS and SHAM conditions.

### 3.2. TTF and Fatigue Index

**Figure 3:**
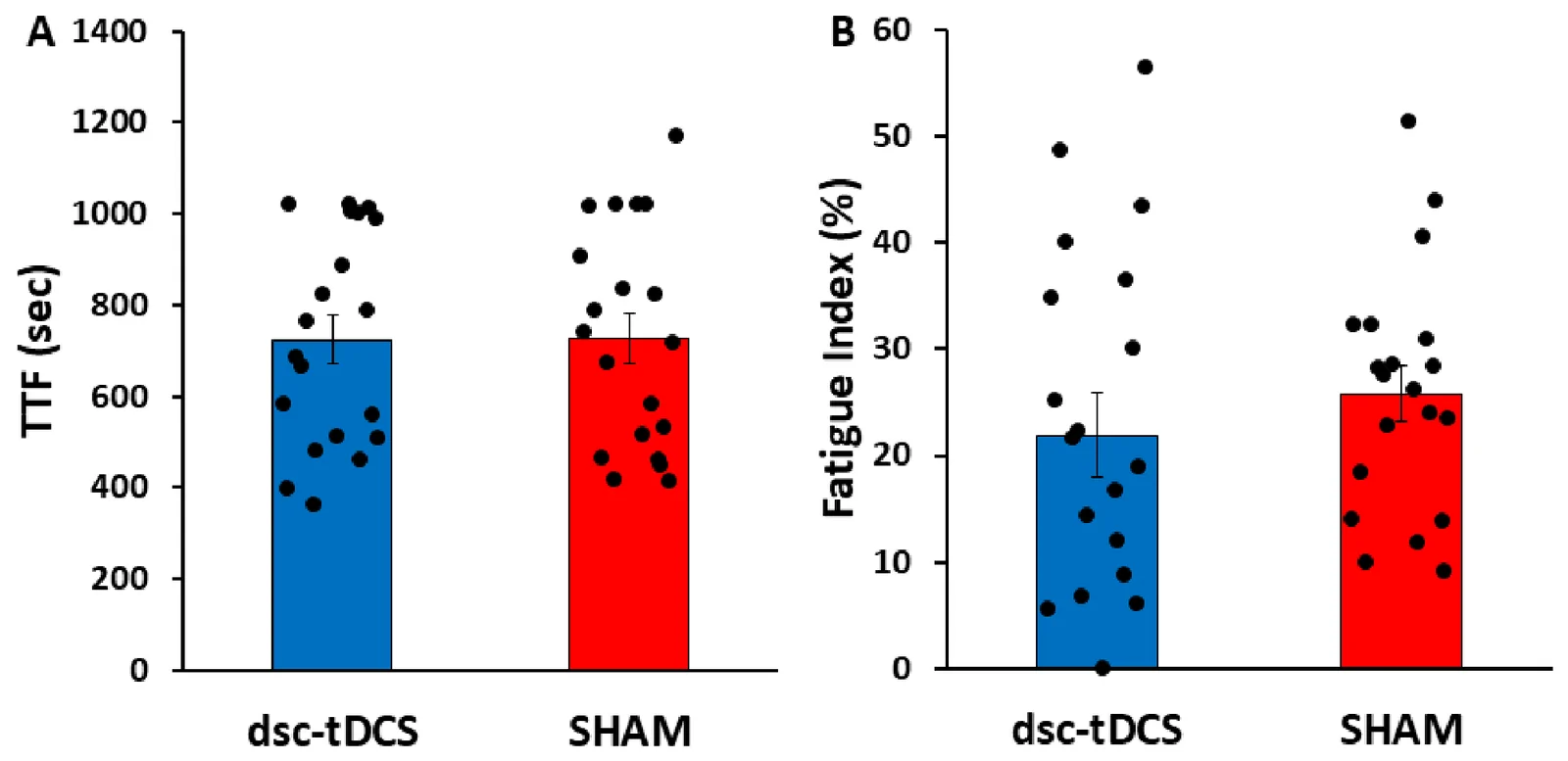
(**A**) The TTF in the fatiguing contraction for the dsc-tDCS and SHAM stimulation conditions. The TTF was nearly identical between the two conditions. Each black dot represents one participant’s TTF in each condition. (**B**) The fatigue index following cessation of the fatiguing contractions for the dsc-tDCS and SHAM stimulation conditions. The percentage of force decline following the fatiguing contraction was comparable between the two conditions. Each black dot represents one participant’s fatigue index in each condition.

The paired *t*-tests indicated that both the TTF (*p* = 0.996; *d* = 0.01; Figure 3A) and the fatigue index (*p* = 0.371; *d* = 0.205; Figure 3B) were not statistically different between the dsc-tDCS and SHAM conditions. The means and standard deviations for these and the other remaining dependent variables are shown in Table 1 below. For TTF, the mixed 2- condition (dsc-tDCS, SHAM) × 2-order (1, 2) ANOVA revealed that the condition main effect (*p* = 0.967; η_p_^2^ = 0.000), order main effect (*p* = 0.811; η_p_^2^ = 0.003), and condition × order interaction (*p* = 0.798; η_p_^2^ = 0.004) were all non-significant. Accordingly, the lack of a significant main effect for order and a condition × order interaction, respectively, rule out the possibility that an order effect or differential order effect influenced the results. Finally, this result indicates that the counterbalancing and washout period achieved their purposes.

### 3.3. aForce and aEMG During Fatigue

**Figure 4:**
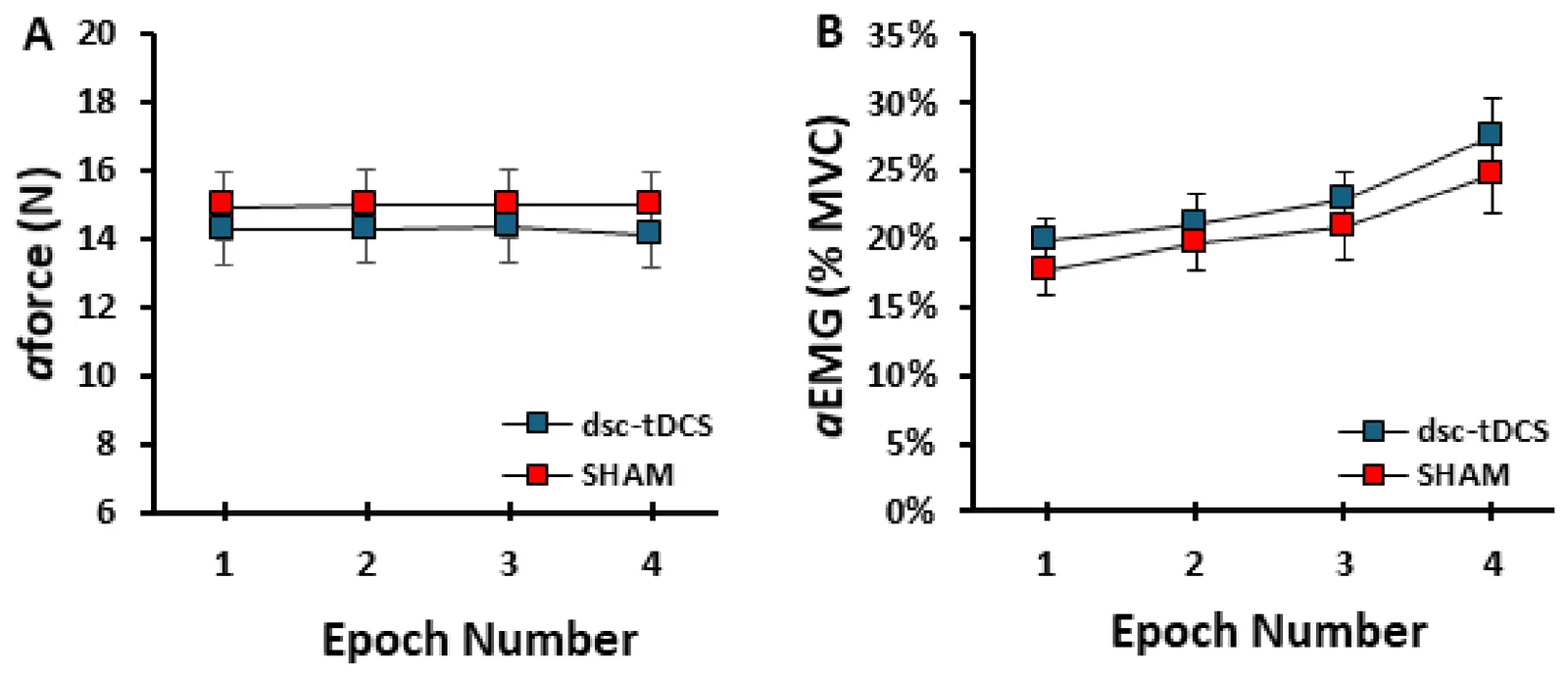
(**A**) The *a*force in each of the four time epochs for the dsc-tDCS and SHAM stimulation conditions. The *a*force was nearly identical across the four time epochs of the fatiguing contraction, which indicated that the participants accurately matched the target force line throughout the contraction until task failure. (**B**) The *a*EMG for the dsc-tDCS and SHAM stimulation conditions in each of the four time epochs. The *a*EMG increased at a comparable rate for the two conditions as a function of epoch number during the fatiguing contraction.

For *a*force, the condition main effect (*p* = 0.203; η_p_^2^ = 0.084), epoch main effect (*p* = 0.241; η_p_^2^ = 0.073), and condition × epoch interaction (*p* = 0.123; η_p_^2^ = 0.106) were all non-significant (Figure 4A). For *a*EMG, the condition main effect (*p* = 0.342; η_p_^2^ = 0.048) and condition × epoch interaction (*p* = 0.741; η_p_^2^ = 0.011) were not significant. However, there was a significant epoch main effect (*p* < 0.001; η_p_^2^ = 0.505) due to the gradual increase in *a*EMG over the course of the fatiguing contractions (Figure 4B). Accordingly, post hoc analysis of the epoch main effect indicated that the *a*EMG activity for epoch 4 was significantly greater compared with epochs 1 (*p* < 0.001), 2 (*p* < 0.001), and 3 (*p* < 0.001). Furthermore, the *a*EMG was significantly greater for epoch 3 compared with epoch 1 (*p* = 0.025).

### 3.4. Force Error and SD of Force During Fatigue

**Figure 5:**
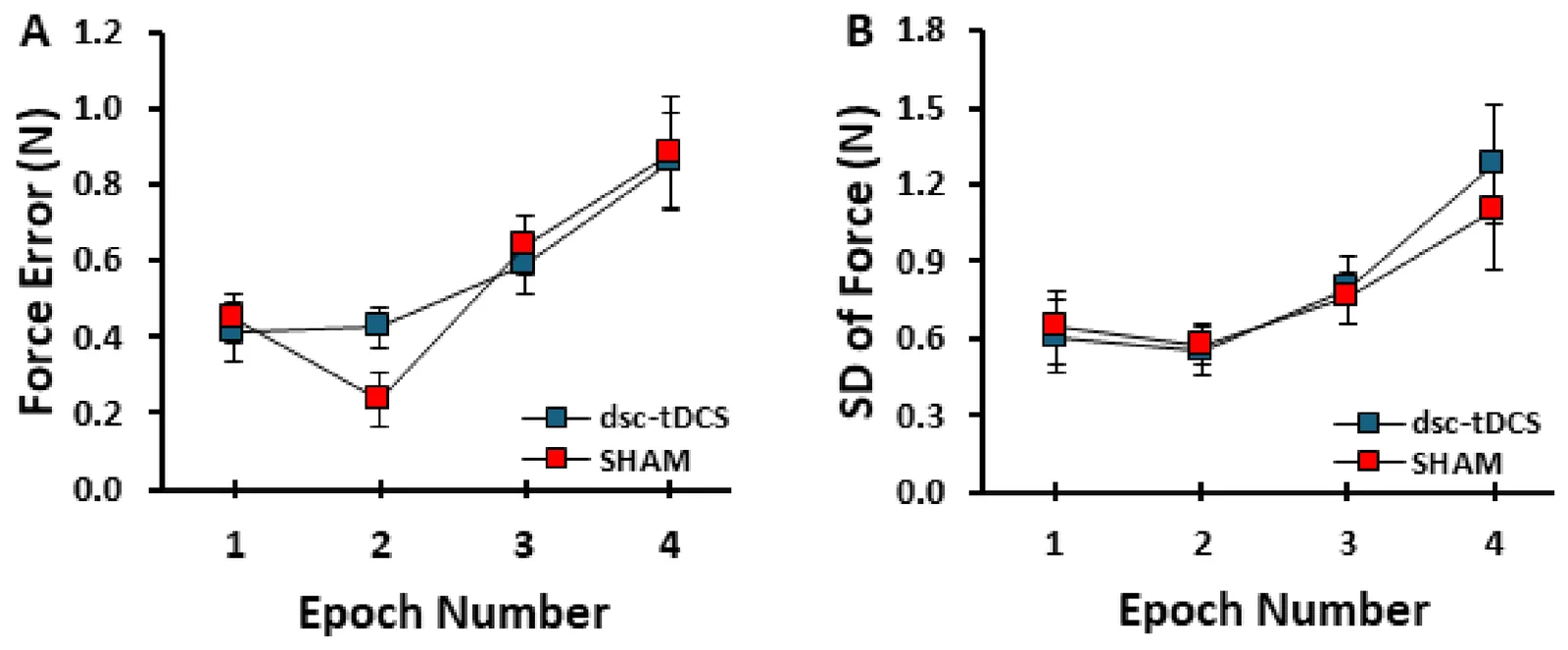
(**A**) The force error in each of the four time epochs for the dsc-tDCS and SHAM stimulation conditions. The force error increased similarly as a function of epoch number for the two conditions. (**B**) The SD of force for the dsc-tDCS and SHAM stimulation conditions in each of the four time epochs. The SD of force increased at a comparable rate as a function of epoch number for the two conditions.

For the force error, the condition main effect (*p* = 0.404; η_p_^2^ = 0.037) and the condition × epoch interaction (*p* = 0.739; η_p_^2^ = 0.012) were both non-statistically significant. However, there was a significant epoch main effect (*p* < 0.001; η_p_^2^ = 0.537) due to the gradual increase in force error over the course of the fatiguing contractions (Figure 5A). Post hoc analysis of the epoch main effect indicated that the force error for epoch 4 was significantly greater than epochs 1 (*p* < 0.001), 2 (*p* < 0.001), and 3 (*p* = 0.006). Furthermore, the force error was significantly greater for epoch 3 compared with epochs 1 (*p* = 0.001) and 2 (*p* < 0.001). In contrast, the force error was similar between epochs 1 and 2 (*p* = 0.987). For SD of force, the condition main effect (*p* = 0.586; η_p_^2^ = 0.016) and the condition × epoch interaction (*p* = 0.409; η_p_^2^ = 0.04) were both non-statistically significant. However, there was a significant epoch main effect (*p* < 0.001; η_p_^2^ = 0.353) due to the gradual increase in SD of force over the course of the fatiguing contractions (Figure 5B). Post hoc analysis of the epoch main effect indicated that the SD of force for epoch 4 was significantly greater than epochs 1 (*p* = 0.016) and 2 (*p* = 0.007). Furthermore, the SD of force was significantly greater for epoch 3 compared with epoch 2 (*p* < 0.001).

### 3.5. 9-HPT Times

**Figure 6:**
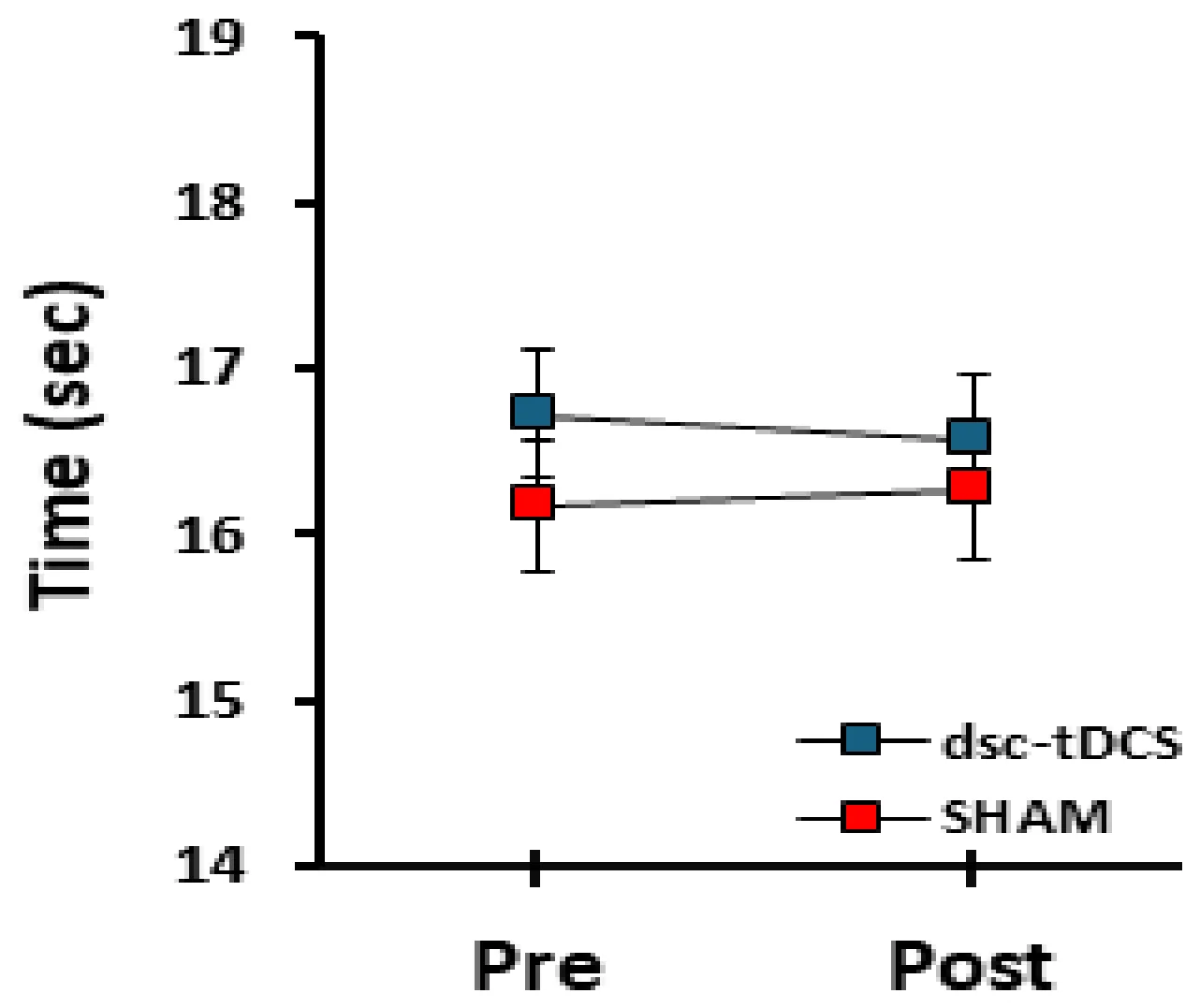
The 9-HPT times for the dsc-tDCS and SHAM stimulation conditions in the pre- and post-tests. The 9-HPT times were similar in both the pre- and post-tests and for the two conditions.

For the 9-HPT times, the main effect for condition (*p* = 0.194; η_p_^2^ = 0.087), the main effect for test (*p* = 0.878; η_p_^2^ = 0.001), and the condition × test interaction (*p* = 0.389; η_p_^2^ = 0.039) were all non-statistically significant (Figure 6).

## 4. Discussion

The purpose of the study was to examine the influence of dsc-tDCS on the TTF of a fatiguing contraction. There were three main findings. First, there were no statistically significant differences in the TTF or the fatigue index between the dsc-tDCS and the SHAM conditions. Second, the rates of increase in FDI *a*EMG activity as well as both the force error and SD of force observed over the course of the fatiguing contraction were also not significantly different between the dsc-tDCS and SHAM conditions. Third, transfer of motor skill did not differ for the two stimulation conditions as indicated by the similar 9-HPT times attained following the fatiguing contraction task. Taken together, the findings indicate that dsc-tDCS does not decrease the rate of progression of muscle fatigue in a sustained submaximal isometric precision grip task in the experimental conditions of the current study.

### 4.1. The Effects of dsc-tDCS on TTF and the Fatigue Index

The current study was the first to focus on the influence of dsc-tDCS in the most common experimental paradigm of a unilateral sustained submaximal isometric fatiguing contraction involving upper limb methods [1,6,9]. Based on the findings of a recent study [38] that reported improved strength and muscle coordination in highly trained gymnasts following application of dsc-tDCS, the original hypothesis was that dsc-tDCS would significantly prolong the TTF of the fatiguing contraction compared to SHAM stimulation. In contrast to this hypothesis, the TTF was nearly identical between the dsc-tDCS and SHAM conditions (percent difference of approximately a quarter of 1%). Similarly, the fatigue index was also extremely similar for the two stimulation conditions and amounted to MVC declines after the fatiguing contraction to approximately 22 and 26% for the dsc-tDCS and SHAM conditions, respectively. Accordingly, neither primary outcome measure approached statistical significance between conditions, and the observed effect sizes were very small. Furthermore, the findings were not due to any potential confounds from differences in the pre-MVCs and the resulting target forces obtained across the two experimental conditions, as these measures also displayed minimal non-statistically significant differences. Furthermore, the *a*force generated in the fatiguing contraction performed in each condition was also nearly the same and remained constant across the four epochs. Therefore, there was neither a systematic nor random difference in the *a*force during the fatiguing contractions, whereby a lower *a*force in one condition could have influenced the TTF values attained. Finally, there was no evidence of an order effect or differential order effect associated with the fully counterbalanced design, which indicates that this potential issue also did not influence the overall findings.

The lack of a significant positive influence of dsc-tDCS on either TTF or the fatigue index is not consistent with most previous studies that involved either DLPFC or M1-tDCS applied during various upper and lower body motor tasks [25,26,64]. The design of the current study and choice of cerebellar tDCS electrode montage, however, were based on the findings of Anoushiravani et al. (2023) [38] who found that dsc-tDCS improved muscle strength and coordination scores as well as EMG activity of a number of upper limb muscles compared to SHAM stimulation. To the best of our knowledge, this study was the first to employ the novel dsc-tDCS electrode montage. Another unique aspect of that study was that it is one of the few tDCS investigations in general that has been conducted using highly trained elite athletes (professional gymnasts) while performing difficult whole-body motor tasks encompassing various aspects of motor performance such as muscle power, force production, and muscle coordination. Accordingly, it is challenging to determine reasons for the dissimilar findings in that study and the current study, but it could be due to the large differences in the motor tasks involved in the two studies.

Although the current findings are in contrast to the balance of the literature, they are in agreement with a number of other studies, which have also failed to find any improvements in metrics of muscle fatigue, including TTF, due to the application of tDCS [25,26,64]. The results of these studies imply that it cannot be presumed that tDCS consistently induces meaningful effects on muscle fatigue resistance. Several related lines of research support this assertion, especially for c-tDCS studies. For instance, a c-tDCS review article [65] recently summarized the results of two research groups that attempted to replicate their initial studies that demonstrated that c-tDCS improved various outcomes of motor function. In both cases, the results were negative and therefore were in contrast to the findings of their initial studies [66,67,68,69]. In addition, a recent study from our laboratory [60] that involved the influence of three days of c-tDCS application on overhand throwing accuracy also failed to replicate a prior single-day c-tDCS [70] overhand-throwing study involving a similar experimental paradigm to the three-day study. Similarly, a prominent fatigue research group performed two different studies involving M1-tDCS [31,71] during sustained submaximal isometric fatiguing contraction conditions. In one study, M1-tDCS was able to improve the TTF of a fatiguing contraction involving the elbow flexors [31], whereas another study [71] performed on the thumb muscles reported that M1-tDCS neither prolonged TTF nor influenced central factors related to the progression of muscle fatigue [71]. In addition, Kenville and colleagues performed two separate c-tDCS studies involving maximal force production in a barbell squat exercise and a barbell bench press exercise [33,72] and found that c-tDCS increased force production in the squat, but not the bench press exercise. Taken together, these five sets of conflicting findings by the same groups of researchers illustrate the heterogeneous nature of M1-tDCS and c-tDCS studies involving both the motor qualities of motor skill and muscle fatigue. Overall, the conflicting results support the assertions of several literature reviews involving tDCS and muscle fatigue [9,25,26,64] that have concluded that any positive influences of tDCS on muscle fatigue are small to moderate and highly variable when the overall literature is considered.

### 4.2. aEMG, Force Accuracy, and Force Variability During the Fatiguing Contraction

The FDI *a*EMG, force error, and SD of force provided measures of muscle activity, force accuracy, and force variability throughout the fatiguing contraction. As expected, there was a progressive increase in all three of these variables as a function of epoch during the fatiguing contraction, while the *a*force remained invariant over the entirety of the fatiguing contraction as the participants correctly kept the force produced centered about the target force line on average. This stereotypical pattern of results is not novel, as it occurs in every sustained submaximal isometric fatiguing contraction study. However, another original hypothesis of the current study was that the rates of increase in *a*EMG, force error, and SD of force would be greater in the SHAM condition, which would lead to a briefer TTF compared with the dsc-tDCS condition. The findings also contradicted that hypothesis, as the measures of *a*EMG activity, force error, and SD of force did increase with time over the course of the fatiguing contractions, but the rates of increase were nearly identical for the dsc-tDCS and SHAM conditions and did not come close to statistical significance. While the physiological mechanisms of lower motor unit discharge rates, augmented motor unit recruitment, and enhanced group III and IV afferent feedback in response to metabolite accumulation [1,3,5,6,73,74,75] were not measured in this study, it can probably be assumed with some degree of confidence that none of these variables were likely to have been different between the two stimulation conditions given the almost identical values attained for TTF, fatigue index, activity, force error, SD of force, and especially *a*EMG. Nonetheless, these physiological mechanisms would have to have been measured directly to draw strong conclusions or make definite comments regarding the details of the possible changes and interactions between these mechanisms in fatiguing contractions completed in the current conditions. Therefore, it should be remembered that different results could potentially be attained in different experimental conditions that could involve different motor tasks, electrode montages, timings of stimulation, and populations.

Relatedly, the three interrelated mechanisms of action whereby tDCS could improve fatigability of increased M1 output (cortical excitability), enhance motor skill, and decrease pain perception were also not likely to have occurred in the dsc-tDCS condition. For example, if dsc-tDCS could lead to an increase in M1 excitability, this could result in acute motor skill increases (lower force error and SD of force) during the fatiguing contraction. Overall, this could increase the TTF by increasing contraction efficacy through the promotion of fewer deviations around the target force line (lower energy expenditure). However, both the force error and SD of force were not influenced by the provision of dsc-tDCS. In addition, dsc-tDCS was also not able to elicit a noticeable transfer of motor skill as indicated by the similar 9-HPT times attained in a fatigued state immediately following the completion of the fatiguing contraction and post-MVCs. Furthermore, any possible modulation of pain perception due to dsc-tDCS was also not likely to have occurred given the complete absence of differences in all of the *a*EMG and force variables. However, it should be remembered that no subjective or objective assessments of exercise-induced muscle pain were carried out in the study. Overall, the current findings suggest that dsc-tDCS did not influence any of the possible physiological mechanisms of action that have been proposed to contribute to the reduction of muscle fatigue progression by tDCS when it occurs, but strong conclusions cannot be drawn regarding many of the aforementioned physiological mechanisms in the current study.

### 4.3. Potential Reasons for the Lack of Ability of dsc-tDCS to Influence Muscle Fatigue

Although the present results were unexpected based on the majority of the c-tDCS literature in general and a previous study involving dsc-tDCS [38], there are a number of possible explanations for the absence of significant results. One likely potential explanation is the choice of motor task and experimental paradigm. Since the cerebellum is most involved in complex movements involving multiple joints and the regulation and exploitation of joint interaction torque, the sustained submaximal isometric contraction task involving hand muscles used in the present study may not have been the most suitable choice for a study involving any form of c-tDCS. Thus, despite the current task conditions being by far the most common for studying muscle fatigue and a reasonable starting point for assessment of the dsc-tDCS montage, in retrospect a more complex motor task could have been more appropriate and should be investigated in future studies. Another possible factor for the lack of dsc-tDCS effects could be related to applying the stimulation during the fatiguing contraction, as opposed to applying the entire duration of the stimulation before the fatiguing contraction, which has been shown to be successful in several studies [25,26,64]. However, other studies have been able to elicit increases in TTF with stimulation undertaken simultaneously with the performance of fatiguing contractions [28,29]. Similarly, the tDCS studies that have reported the greatest magnitude of motor learning improvements involved application of the tDCS during motor task practice [18,19,20]. A final possibility is the presence of ceiling effects due to the fact that this study recruited only active young adult participants. Accordingly, a series of tDCS motor skill investigations [76,77,78,79] have attributed negative findings to the level of skill proficiency of the performer or the initial skill level of individual participants in the overall study population.

### 4.4. Study Limitations

There were several inherent limitations of the study that should be acknowledged and briefly discussed. First, a different c-tDCS electrode montage may have been more effective for delaying the progression of fatigue than the dsc-tDCS electrode montage. In particular, the typical c-tDCS montage utilized successfully in motor skill studies by other research groups [39,45,67,80,81,82,83,84] and in a previous study in our laboratory [70] would be an obvious choice, although other recent studies have failed to demonstrate significant increases in motor skill using this electrode montage [60,68,69]. Another alternative would be the bilateral c-tDCS montage utilized by Kenville and colleagues (2020) [33], although it is somewhat difficult to see how this electrode montage would yield different results compared to the electrode montage used in the current study and by Anoushiravani et al. (2023) [38]. Second, application of dsc-tDCS prior to the fatiguing contraction [26,27,32,85,86] has been successful in previous studies. Thus, this timing could be more efficacious than delivering dsc-tDCS during the fatiguing contraction. However, other tDCS fatigue studies have reported positive effects with concurrent stimulation [28,29], and the most successful tDCS motor skill studies were also performed simultaneously with motor practice [18,19,20,81]. Third, some research has suggested that higher stimulation intensities of 4 mA could lead to greater positive effects, although a few available studies have not been able to demonstrate clear benefits of this current strength on muscle fatigue [87,88]. Fourth, the details of the motor task could have precluded the ability of dsc-tDCS to enhance fatigue resistance, and motor tasks characterized by high force contractions may have a greater probability of responding to c-tDCS [33] or M1-tDCS based on some tDCS strength training studies [89,90,91,92,93,94]. Most importantly, it is possible that a more complex motor task involving more coordination, multiple joints, and anisometric contractions as opposed to isometric contractions would have been more likely to yield significant results. Nonetheless, another recent c-tDCS study by Kenville and colleagues (2024) [72] using the same bilateral c-tDCS montage with a single anode as their previous barbell squat study [33] failed to augment MVC force or rate of force development in an isometric barbell bench press. Fifth, the study only involved basic force and EMG measures. Ideally, TMS measures of net cortical excitability (motor evoked potential amplitude) and intracortical interneuronal pathways such as short interval intracortical inhibition (SICI) and intracortical facilitation (ICF) [95,96] would have been valuable along with measures of voluntary activation, to name a few possibilities. However, these measures were well beyond the scope of this initial study, which mainly sought to determine if dsc-tDCS could improve the motor performance variable of fatigue resistance. If dsc-tDCS had been successful in this study, follow-up studies would have been planned to investigate the underlying mechanisms in detail. Sixth, the study did not utilize any of the various questionnaires that are sometimes used in tDCS studies and have been recommended in review articles [97,98], such as those evaluating blinding integrity, ratings of perceived exertion, pain, motivation, or subject tolerance. Therefore, formal assessments of blinding effectiveness and participant subjective perceptions were not performed, which could have influenced results. Finally, the study did not evaluate the interrelated issues of how the possible differences in individual anatomy [65] could have influenced the total amount of current delivered to the cerebellum and the details of the distribution of the current, even though a previously validated montage was used. It is possible that individualizing the current level and using neuronavigation to perhaps better optimize the exact positioning of the electrodes for each subject could have led to different outcomes. All of these issues will have to be addressed in greater detail in future more elaborate studies to determine the viability of dsc-tDCS as an intervention to influence muscle fatigue.

## 5. Conclusions

In summary, the application of dsc-tDCS did not significantly increase the TTF or influence the fatigue index compared to the SHAM condition. Furthermore, the rates of increase in measures of fatigue progression, including *a*EMG, force error, and SD of force recorded during the fatiguing contraction, were also not significantly different between the dsc-tDCS and SHAM stimulation conditions. Finally, transfer of motor skill in the fatigued state, as indicated by 9-HPT times obtained following the fatiguing contraction, was also similar for the two stimulation conditions. These findings are not consistent with the only available prior study involving the dsc-tDCS electrode montage [38]. Collectively, these results suggest that dsc-tDCS is not an effective intervention to mitigate fatigue development, at least in the task conditions of the current study, as no statistically significant effects were observed. Accordingly, these findings do not necessarily argue against cerebellar tDCS as a therapeutic approach in general. Instead, they suggest that this specific bilateral dual source montage does not appear to improve fatigue resistance in healthy young adults performing this particular precision grip fatigue task. Therefore, future research is appropriate to further explore the effects of dsc-tDCS and other c-tDCS electrode montages on various motor tasks that involve motor skill, maximal force production, or fatiguing exercise.

## Figures and Tables

**Table 1:** Mean and (SD) of the primary and selected secondary dependent variables in the dsc-tDCS and SHAM conditions.

Condition/Epoch	TTF (s)	Fatigue Index (%)	*a*force (N)	*a*EMG (% MVC)	Force Error (N)	SD of Force (N)	9-HPT (s)
dsc-tDCS	724.80 (233.52)	21.95 (17.18)	-	-	-	-	-
Pre	-	-	-	-	-	-	16.73 (1.70)
Post	-	-	-	-	-	-	16.58 (1.71)
Epoch 1	-	-	14.27 (3.91)	19.93 (6.85)	0.41 (0.35)	0.61 (0.63)	-
Epoch 2	-	-	14.32 (3.79)	21.19 (9.30)	0.43 (0.23)	0.55 (0.42)	-
Epoch 3	-	-	14.32 (3.82)	22.85 (9.26)	0.59 (0.34)	0.79 (0.58)	-
Epoch 4	-	-	14.13 (3.80)	27.49 (12.82)	0.86 (0.55)	1.28 (1.00)	-
SHAM	726.55 (241.92)	25.8 (11.25)	-	-	-	-	-
Pre	-	-	-	-	-	-	16.17 (1.73)
Post	-	-	-	-	-	-	16.26 (1.81)
Epoch 1	-	-	14.97 (4.36)	17.69 (8.30)	0.45 (0.28)	0.64 (0.63)	-
Epoch 2	-	-	15.02 (4.38)	19.66 (8.92)	0.51 (0.31)	0.58 (0.35)	-
Epoch 3	-	-	15.01 (4.38)	20.95 (10.59)	0.64 (0.34)	0.76 (0.44)	-
Epoch 4	-	-	14.99 (4.47)	24.65 (12.21)	0.88 (0.63)	1.10 (1.02)	-

## Data Availability

The data presented in this study are available on request from the corresponding author.
